# Evaluating the Feasibility and Preliminary Effectiveness of a Tailored Diabetes Education Program for Rural Residents

**DOI:** 10.13023/jah.0801.06

**Published:** 2026-04-01

**Authors:** Brittany L. Smalls, Oluwatosin Leshi, Suraksha Khanal, Grejika E. Abram-Erby

**Affiliations:** University of Texas Medical Branch; University of Texas Medical Branch; University of Texas Medical Branch; University of Kentucky

**Keywords:** Appalachia, diabetes, diabetes education, self-care, self-management, social determinants of health, social support, socioenvironmental factors, T2D, tailored intervention

## Abstract

**Introduction:**

Diabetes is a top ten Rural Healthy People 2030 priority for health improvement among rural Americans. Socioeconomic, environmental, geographic and political barriers contribute to the type 2 diabetes (T2D) prevalence and its disparate impact in rural communities. However, promoting proper self-care behaviors via self-management education can help people with diabetes better manage their condition.

**Purpose:**

To account for the social environmental factors that affect rural residents, a tailored diabetes education program was developed, and the program’s effectiveness in improving diabetes knowledge and T2D-related health outcomes was assessed. Methods: Using data collected from Appalachian Kentucky residents enrolled in a six-week, tailored educational intervention, this study used linear mixed models to assess the associations with clinical outcomes, knowledge, self-care, attitudes, and social support post intervention.

**Results:**

Eighteen respondents consented to participate in the study (mean age: 58.3 years). Diabetes knowledge and attitudes improved significantly at six months post intervention. Positive changes in social support were also noted in that time frame. Self-care behaviors remained consistent post intervention, yet key metabolic outcomes (e.g., HbA1c, triglycerides, and BMI) decreased significantly.

**Implications:**

Few studies have examined the effectiveness of a self-management intervention tailored for older, rural-dwelling Kentuckians living with T2D. With these promising results, tailored interventions that incorporate social support and social determinants of health hold the potential to improve the health and quality of life of adults with diabetes, paving the way for innovative and sustainable approaches to diabetes care.

## INTRODUCTION

Recent prevalence data estimates that 38.4 million people are affected by diabetes.^1^ The prevalence of type 2 diabetes (T2D) is about 17% higher in rural areas than in urban areas.^2^ This imbalance is accentuated in Kentucky, where the T2D prevalence is 16.1% in rural Appalachian counties, while the overall state prevalence is 13.8%.^3^ Without intervention, morbidity and mortality associated with diabetes will continue to devastate this vulnerable population.^4^ Socioeconomic factors predict T2D prevalence and contribute to its unequal impact in rural communities,^1^,^2^ which are evident via economic factors, social norms, and political systems that impact rural communities, hence the need to provide tailored resources in rural communities that align with reducing health disparities and improving outcomes.

Diabetes education is a critical component of equipping those diagnosed with T2D with the knowledge needed to manage their disease. Educational resources, such as diabetes self-management training or education (DSMT/DSME), are often unavailable in rural communities. In the rare instances that these educational opportunities are available, the materials are not adapted to surmount the socioenvironmental challenges that may impact their ability to engage in healthful behaviors.^5^ Therefore, the purpose of this feasibility study was to account for the social environmental factors that affect rural residents in a tailored diabetes education program and to examine the program’s effectiveness in improving diabetes knowledge and T2D-related health outcomes.

## METHODS

### Study Sample and Intervention

Participants were recruited using convenience sampling from the University of Kentucky Barnstable Brown Diabetes and Obesity Center from 2021 – 2022. Eligible participants were 18+ years old, had a confirmed diagnosis of T2D, could understand and speak English, and resided in a rural Kentucky county. Participants who met the criteria consented and were enrolled in the study. After baseline information was obtained, participants completed a six-week-long, tailored diabetes education intervention. The sessions were 90 minutes long and covered self-care, diabetes and insulin resistance, sugar-sweetened dangers, fats and proteins that heal, oxidative stress, and epigenetics. The first session was conducted in person to obtain consent and collect baseline data. Participants had the option to continue to attend sessions in person or virtually (e.g., Zoom, Skype, telephone). All study procedures were approved by the University of Kentucky Institutional Review Board (protocol #63765).

### Data Collection

Data were collected at baseline, three months, and six months post-intervention. Baseline data was collected after consent and included demographics, validated questionnaires, and clinical outcomes (e.g., A1c, blood pressure, lipids), along with height, weight, and waist circumference. Blood pressure was measured using a cuff and blood glucose via the fingerstick method; lipids were extracted from electronic medical records. Demographic characteristics included age, sex, race, ethnicity, educational status, insurance, and employment status. Validated surveys used were the Medical Outcomes Study Social Support Survey,^6^ EuroQol-5D,^7^ Diabetes Self-Management Questionnaire,^8^ Diabetes Knowledge Questionnaire,^9^ and the Diabetes Empowerment Scale.^10^ Change in A1c was the primary outcome, whereas changes in other clinical measures, diabetes knowledge, and psychosocial factors were secondary outcomes.

### Statistical Analysis

Sample characteristics were calculated for the overall sample. Continuous variables were reported as means and standard errors, while categorical variables were reported as frequencies and proportions in each category. We used linear mixed models (LMM) to examine the effect of time on HbA1c, diabetes knowledge, diabetes self-care management, diabetes attitude, and clinical outcome. Time was included as a categorical fixed effect, and a random intercept for each participant was included to account for within-subject correlation. For primary analysis, linear mixed model was performed to assess the association between HbA1c and diabetes knowledge, diabetes self-care, diabetes empowerment, and time point. We then fit two sets of models: (1) Unadjusted; (2) adjusted for age, race, gender, employment, education, and insurance. Lastly, all parameter estimates were expressed with 95% confidence interval and were considered statistically significant if *p* < 0.05. Analyses were conducted in SAS (version 9.4).

## RESULTS

### Participant Characteristics

Table 1 displays the sample characteristics of the study population. A total of 18 participants consented to the study, with a mean age of 58 years. Most of the respondents in this sample were female (55.5%), non-Hispanic white (73.3%), insured (94.4%), and employed (66.6%). Approximately 44.4% of respondents had some education beyond high school. Three-quarters of the sample were white, and all respondents (100%) reported their ethnicity as not Hispanic or Latino. Out of 18 study participants, 16 participants had participated in all study sessions, among which two participants had in-person sessions and 11 had virtual sessions.

### Primary Analysis

The descriptives for HbA1c, diabetes knowledge, self-care behavior, diabetes empowerment, weight, and body mass index can be found in Table 2. Diabetes knowledge (LS means = 2.50, Cl = 0.47, 4.52, *p* = 0.01) and diabetes self-care (LS means = 5.31 Cl = 1.98, 8.64, *p* = 0.001) for three months was significantly higher than baseline, whereas diabetes attitude showed no significant difference between baseline and three months (LS means = −9.48, Cl = −27.49, 8.52, *p* = 0.39) (Table 3). Diabetes knowledge, diabetes self-care, and diabetes attitude showed significant differences in means from baseline to six months. Linear mixed effects model was used to measure self-care, diabetes knowledge, diabetes empowerment, and HbA1c associations. Self-care was negatively associated with HbA1c (Table 4; β [Confidence interval]: −0.05 [−0.15, 0.03]). This association showed that each point increase in self-care management was associated with a decrease in HbA1c levels by 0.05. Similarly, for diabetes knowledge, decreases in knowledge were associated in increases in HbA1c levels (β= −0.05, CI = −0.14-0.03). This assumption was also true for diabetes attitude, where higher scores in attitude were associated with low levels of HbA1c (β= −0.007, CI = −0.02-0.01).

## DISCUSSION

Over the past two decades, diabetes prevalence has risen significantly in the United States (U.S.), with rates increasing particularly among older adults.^11^,^12^ Recent national data indicate an average age of 61.1 years for those diagnosed with diabetes,^13^ suggesting our sample’s age distribution (mean 58.3 years) is reflective of broader trends in diabetes demographics.^13^,^14^ Patients with more than a high school education have lower prevalence and risk of diabetes compared to those with less education.^15^,^16^ Contrastingly, in our study, most participants had education levels higher than high school. The inverse relationship between diabetes diagnosis and educational level could be attributed to existing knowledge gaps in underserved communities,^17^ where individuals, particularly older adults, may lack adequate understanding of diabetes management practices, which potentially impact self-management behaviors.^18^–^20^

Diabetes is inherently challenging to manage, requiring individuals to draw on knowledge, motivation, and self-management skills to achieve desired health outcomes.^21^ Previous studies have implicated diabetes knowledge in better diabetes self-care management behaviors.^22^–^24^ Our study found significant improvements in diabetes knowledge and attitudes, emphasizing the importance of education in fostering informed decision-making for self-management. The improvement in diabetes knowledge (mean increase of 2.62, *p* =0.004) suggests our intervention effectively enhanced participants’ understanding, which likely reflects the appropriateness of our tailored and interactive educational methods. This finding is consistent with earlier studies, which highlighted the benefits of personalized diabetes education for knowledge improvement.^25^ Improved knowledge is foundational to long-term diabetes management, yet translating this knowledge into sustained behavioral change remains challenging. This finding is consistent with the study by Sebire et al., which highlights how intrinsic motivation fosters more sustainable behavior change over time.^26^

Our feasibility study led to significant improvements in several key metabolic parameters. Most notably, a 0.41% drop in HbA1c aligns with outcomes seen in similar lifestyle interventions.^27^–^30^ The intervention also resulted in a notable decrease in triglyceride levels, showing consistent results with an earlier study.^60^ These positive changes in triglycerides are meaningful given their role in cardiovascular risk, which is especially relevant for individuals managing T2D. Reduction in participants’ weight and BMI further reinforces the potential for lifestyle interventions to improve metabolic health. Our study is consistent with extensive research showing the association between weight loss and improved glycemic control.^30^–^33^ The trending improvement of metabolic outcomes in this study likely reflects the tailored program — which included dietary education, physical activity promotion, and psychological supports relevant to rural residents’ socioenvironmental experience — and the structured yet flexible delivery methods. Overall, these results are consistent with a systematic review evaluating tailored diabetes education interventions for rural residents, showing improvements in clinical, knowledge, and psychosocial outcomes.^34^ The review also supports the use of online and virtual options for tailored diabetes education for rural residents.^34^

### Limitations

This feasibility study presents potential limitations that warrant consideration. The small sample size limits the generalizability of findings and reduces the statistical power to detect significant effects. The short follow-up period may not capture the sustainability of behavioral and metabolic improvements, and the sample lacks diversity and restricts applicability to broader populations.

## CONCLUSION

Randomized controlled trials with follow-ups beyond six months can provide insights into the sustainability of the improvements and the factors that foster long-term adherence. Tailored interventions alongside community-based support systems can also improve accessibility and appeal to diverse populations. Expanding studies to include larger and more diverse samples will enhance the generalizability of findings and clarify intervention effectiveness across varied settings.

**SUMMARY BOX**

**What is already known about this topic?**

Kentucky’s Appalachian counties have some of the highest T2D prevalence rates. Proper self-care practices can help reduce the challenges and adverse outcomes associated with T2D and help individuals manage the disease on their own.

**What is added by this report?**

This manuscript may provide one of the first assessments of a tailored, T2D self-management intervention for rural-dwelling individuals. Following the intervention, respondents saw improved diabetes knowledge and attitudes, social support, and metabolic outcomes. Overall self-care behaviors also remained stable through follow up.

**What are the implications for future research?**

The findings offer a framework for innovative and sustainable interventions that can improve accessibility and health outcomes beyond rural Appalachian populations to other underserved communities, thus appealing to diverse populations and diverse needs. Such actions may underscore the generalizability of the findings and the effectiveness of the intervention.

## Figures and Tables

**Table 1:** Sample Characteristics (N=18)

Variables	N (%) or mean (SD)
**Age** a	58.25(6.3)
**Gender**	
Male	8(44.4)
Female	10(55.6)
**Race**	
Not Hispanic white	12(75.0)
Black or African American	4(26.6)
**Sexual Orientation**	
Bisexual	1(5.9)
Gay	2(11.8)
Heterosexual or straight	14(82.3)
**Education**	
Some high school	1(5.6)
High school diploma	1(5.6)
Some education beyond high school but no degree	8(44.4)
College degree	6(33.3)
Graduate or professional school but no advance degree	0
Graduate or professional degree	2(11.1)
**Insurance Status**	
Yes	17(94.4)
No	1(5.6)
**Employment**	
Yes	12(66.7)
No	6(33.3)
**Weight** a	218.53 (48.22)
**Waist Circumference** a	110.38 (19.11)
**Height** a	66.30 (4.62)

NOTES:

a Continuous variables reported as mean and standard error, while other reported as count and percent.

**Table 2:** Scores and Descriptives for Diabetes Knowledge, Self-Care, Diabetes Attitudes, and Clinical Outcomes, and Social Support

*Assessment of Diabetes Knowledge, Self-Care, and Attitude*				
Variables	Baseline Mean (SD)^b^	3-month Mean (SD)^b^	6-month Mean (SD)^b^	*p*-value
**Diabetes Knowledge Questionnaire** a	10.5 (2.6)	12.57 (1.8)	13.16 (0.9)	0.004^c^, 0.02^d^, 0.44^e^
***Diabetes Self-Management***				
Sum scale^a^	30.6 (6.87)	37.0 (4.28)	36.0 (5.93)	0.003^c^, 0.0006^d^ 0.466^e^
Glucose monitoring	10.44 (3.18)	12.0 (2.40)	11.50 (2.96)	0.32^c^, 0.02^d^, 0.17^e^
Physical activity	4.40 (2.85)	6.63 (1.21)	6.25 (2.30)	0.01^c^, 0.002^d^, 0.49^e^
Dietary control	5.75 (1.77)	7.08 (1.62)	7.08 (2.50)	0.03^c^, 0.04^d^, 1.00^e^
Health care usage	7.81 (1.64)	9.0 (0)	8.58 (0.99)	0.61^c^, 0.04^d^, 0.11^e^
**Diabetes Attitude Questionnaire** a	61.47(15.99)	52.0(29.20)	43.33(12.83)	0.02^c^, 0.19^d^, 0.27^e^
***Assessment of Clinical Outcomes***				
**Weight**	218.52(40.04)	206.90(41.6)	208.47(44.9)	0.19^c^, 0.11^d^, 0.76^e^
**Height**	66.30(4.62)	65.75(3.98)	65.81 (4.1)	0.51^c^, 0.13^d^, 0.41^e^
**BMI**	34.85(4.85)	33.55 (5.73)	33.98 (6.60)	0.24^c^, 0.10^d^, 0.62^e^
**SBP**	124.94(13.44)	124.25 (16.04)	126.08 (14.04)	0.82^c^, 0.83^d^, 0.68^e^
**DBP**	77.47(9.2)	78.08 (11.54)	79.58 (5.82)	0.56^c^, 0.98^d^, 0.57^e^
**HbA1c**	7.68(1.3)	7.49 (1.04)	7.28 (0.91)	0.34^c^, 0.81^d^, 0.24^e^
**Total cholesterol**	145.60(40.4)	153.81 (45.00)	150.8 (47.7)	0.67^c^, 0.24^d^, 0.48^e^
**LDL**	71.52(30.23)	74.14(41.9)	75.78(44.13)	0.67^c^, 0.67^d^, 0.99^e^
**HDL**	43.40(15.03)	44.54 (14.4)	44.5 (15.25)	0.19^c^, 0.11^d^, 0.81^e^
**Triglycerides**	187.46(101.4)	162.81(92.7)	152.6 (58.82)	0.04^c^, 0.31^d^, 0.27^e^
***Assessment of Social Support***				
**Social Support Summary Score** a	**60.33 (23.99)**	**66.25 (22.9)**	**71.09 (23.00)**	0.08^c^, 0.34^d^, 0.36^e^
**Emotional support** a	**25.80(10.2)**	**27.41(10.1)**	**28.53(10.6)**	0.19^c^, 0.69^d^, 0.34^e^
None of the time	3(16.7)	1(7.7)	0	
A little of the time	4(22.2)	2(154)	3(25.00)	
Some of the time	4(16.7)	3(23.1)	1(8.33)	
Most of the time	4(22.2)	3(23.1)	3(25.00)	
All the time	4(22.2)	4(30.8)	5(41.7)	
**Affectionate support** a	**10.68(4.8)**	**11.16(4.9)**	**10.91(4.6)**	0.83^c^, 0.60^d^, 0.76^e^
None of the time	5(27.8)	2(15.4)	0	
A little of the time	1(5.6)	2(15.4)	3(25.0)	
Some of the time	2(11.11)	1(7.7)	2(16.7)	
Most of the time	2(11.11)	2(15.4)	1(8.3)	
All of the time	8(44.44)	6(46.2)	6(50.0)	
**Tangible support** a	**11.87(5.81)**	**13.50(5.00)**	**13.91(5.90)**	0.32^c^, 0.55^d^, 0.69^e^
None of the time	3(16.7)	1(7.7)	0	
A little of the time	6(33.33)	2(15.4)	2(16.7)	
Some of the time	2(11.11)	3(23.1)	5(41.7)	
Most of the time	3(16.7)	4(30.8)	0	
All the time	4(22.22)	3(23.1)	5(41.7)	
**Positive Social Interaction** a	**9.18(4.3)**	**10.66(4.4)**	**11.45(3.32)**	0.12 c, 0.24 d, 0.66^e^
None of the time	4(22.2)	2(15.4)	1(8.33)	
A little of the time	5(27.8)	2(15.4)	1(8.33)	
Some of the time	2(11.11)	1(7.7)	3(25.0)	
Most of the time	3(16.7)	3(23.1)	3(25.0)	
All the time	4(22.22)	5(38.5)	4(33.33)	

NOTES:

a Composite social support summary score calculated as the sum of the responses from the listed questions such that higher value indicates a more positive social support.

b Mean and standard error of all responses for a given question are reported on rows where questions are specified. Count and % are given for individual response options.

c p-values for baseline to 6 months

d p-values for baseline to 3 months

e p-values for 3 months to 6 months

**Table 3:** Descriptives of Quality of Life

*Assessment of Quality of Life*				
Variables	Baseline Mean (SD) or Count %)^b^	3-month Follow Up Mean (SD) or Count %)^b^	6-month Follow Up Mean (SD) or Count %)^b^	*p*-value*
**Pain/Discomfort**				N/A
No problem	4(25.0)	3(25.0)	4(33.33)	
Slight problem	6(37.50)	3(25.0)	4(33.33)	
Moderate problem	4(25.0)	5(41.67)	3(25.0)	
Unable	2(12.50)	1(8.33)	1(8.33)	
**Anxiety/Depression**				N/A
No problem	7(43.75)	7(58.33)	6(50.0)	
Slight problem	5(31.25)	2(16.67)	4(33.33)	
Moderate problem	3(18.75)	2(16.67)	2(16.67)	
Unable	1(6.25)	1(8.33)	0	
**Usual Activities**				N/A
No problem	8(50)	5(41.67)	6(20.0)	
Slight problem	4(25)	6(50.0)	4(33.33)	
Moderate problem	3(18.75)	1(8.33)	2(16.67)	
Unable	1(6.25)	0	0	
**Self-Care**				N/A
No problem	14(87.50)	11(91.67)	11(91.67)	
Slight problem	2(12.50)	1(8.33)	0	
Moderate problem	0	0	1(8.33)	
**Mobility**				N/A
No problem	6(37.50)	4(33.33)	5(41.67)	
Slight problem	6(37.50)	6(50)	4(33.33)	
Moderate problem	4(25.00)	2(16.67)	3(25.0)	
**Overall, Health Today**	69.38(20.44)	65.81(20.53)	67.91(24.60)	0.91^c^, 0.64^d^, 0.72^e^

NOTES:

a Composite social support summary score calculated as the sum of the responses from the listed questions such that higher value indicates a more positive social support.

b Mean and standard error of all responses for a given question are reported on rows where questions are specified. Count and % are given for individual response options.

c p-values for baseline to 6 months

d p-values for baseline to 3 month

e p-values for 3 months to 6 month

* p-values were not included for the individual items due to low response values, which could yield unreliable interpretations of the data

**Table 4:** Association of Hba1c with Diabetes Knowledge, Diabetes Self-Management, and Attitude

Variables of interest	Estimate (β), 95% CL	p-value
Diabetes knowledge	−0.05 [−0.14, 0.03]	0.20
Diabetes self-care management	−0.05 [−0.15, 0.03]	0.18
Attitude towards diabetes	−0.007 [−0.02, 0.01]	0.37
